# Holmium(III) Single-Ion Magnet for Cryomagnetic Refrigeration Based on an MRI Contrast Agent Derivative

**DOI:** 10.1021/acs.inorgchem.1c01905

**Published:** 2021-08-23

**Authors:** Borja Rodríguez-Barea, Júlia Mayans, Renato Rabelo, Adrián Sanchis-Perucho, Nicolás Moliner, José Martínez-Lillo, Miguel Julve, Francesc Lloret, Rafael Ruiz-García, Joan Cano

**Affiliations:** ‡ Instituto de Ciencia Molecular/Departament de Química Inorgànica, Facultat de Química, Universitat de València, Paterna, València 46980, Spain

## Abstract

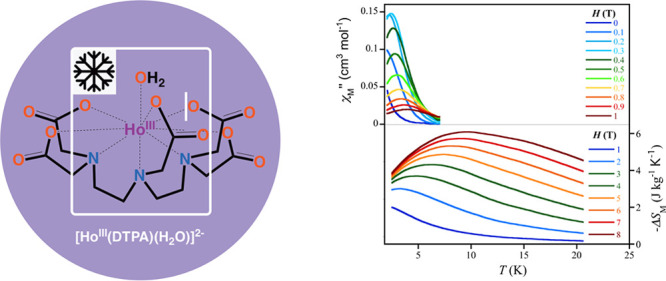

The coexistence of field-induced blockage of the magnetization and significant magnetocaloric effects in the low-temperature region occurs in a mononuclear holmium(III) diethylenetriamine-*N*,*N*,*N*′,*N*″,*N*″-pentaacetate complex, whose gadolinium(III) analogue is a commercial MRI contrast agent. Both properties make it a suitable candidate for cryogenic magnetic refrigeration, thus enlarging the variety of applications of this simple class of multifunctional molecular nanomagnets.

## Abstract 2

A mononuclear holmium(III) complex is proposed as a new prototype of a multifunctional molecular nanomagnet for the physical implementation of cryomagnetic refrigeration near the strategically relevant hydrogen liquefaction temperature. Significant magnetocaloric effects occur in a wide temperature range just above the field-induced magnetization blockage.

Mononuclear complexes of lanthanide(III) ions exhibiting slow relaxation of the magnetization, so-called lanthanide single-ion magnets (SIMs), have attracted much attention in diverse areas of nanoscience and nanotechnology.^1−7^ Because of their well-known magnetothermal and quantum coherence properties, mononuclear gadolinium(III), dysprosium(III), and holmium(III) derivatives are illustrative examples of the promising applications of Ln SIMs as both molecular cryomagnetic coolants and qubits for cryogenic magnetic refrigeration (CMR) and quantum information processing technologies.^8−11^ In the search for molecular materials that exhibit various physical properties linked to technological applications, choosing a starting molecule with one of these properties should be a good strategy.

In this context, we have turned our eyes toward the well-known family of mononuclear complexes with Ln^III^ ions (Ln = Gd, Dy, and Ho) and either linear or cyclic polyaminocarboxylate ligands, widely investigated as magnetic resonance imaging (MRI) contrast agents (Scheme S1).^12,13^ Herein we report our first results along this line covering the synthesis, structural, and general physicochemical characterization of the novel holmium(III) diethylenetriamine-*N*,*N*,*N*′,*N*″,*N*″-pentaacetate (DTPA) complex of the formula Na_2_[Ho^III^(DTPA)(H_2_O)]·8H_2_O (**1**), as well as a preliminary investigation of the magnetic field dependence of its magnetic and magnetothermal properties through direct-current (dc) and alternating-current (ac) magnetic measurements. Compound **1** constitutes a unique example of Ho^III^ SIM that would operate as a cryomagnetic coolant close enough to the strategically relevant hydrogen liquefaction temperature.

**1** was prepared from the stoichiometric reaction of H_5_DTPA and holmium(III) oxide in water under reflux for several hours after neutralization with NaHCO_3_ until pH ca. 6.5 (see the experimental section and Figures S1–S3). X-ray-suitable pale-pink prisms were obtained after several weeks of slow evaporation at room temperature. It crystallizes in the *P*2_1_/*n* space group of the monoclinic system (Table S1). The asymmetric unit consists of two sodium(I)-bridged dianionic mononuclear [Ho^III^(DTPA)(H_2_O)]^2–^ units, as shown in Figure 1. The two crystallographically independent nine-coordinate Ho1 and Ho2 atoms exhibit similar spherically distorted geometries intermediate between a tricapped trigonal prism (TCTPR) and a monocapped square antiprism (CSAPR) of *D*_3*h*_ and *C*_4*v*_ symmetry (Scheme S2 and Table S2).^14,15^ The values of the shape measures for the Ho1/Ho2 atoms are *S*(TCTPR) = 1.150/1.000 and *S*(CSAPR) = 1.083/1.089 (*S* = 0 for a perfect match with the ideal polyhedron), supporting the previous conclusion.^16^

**Figure 1:**
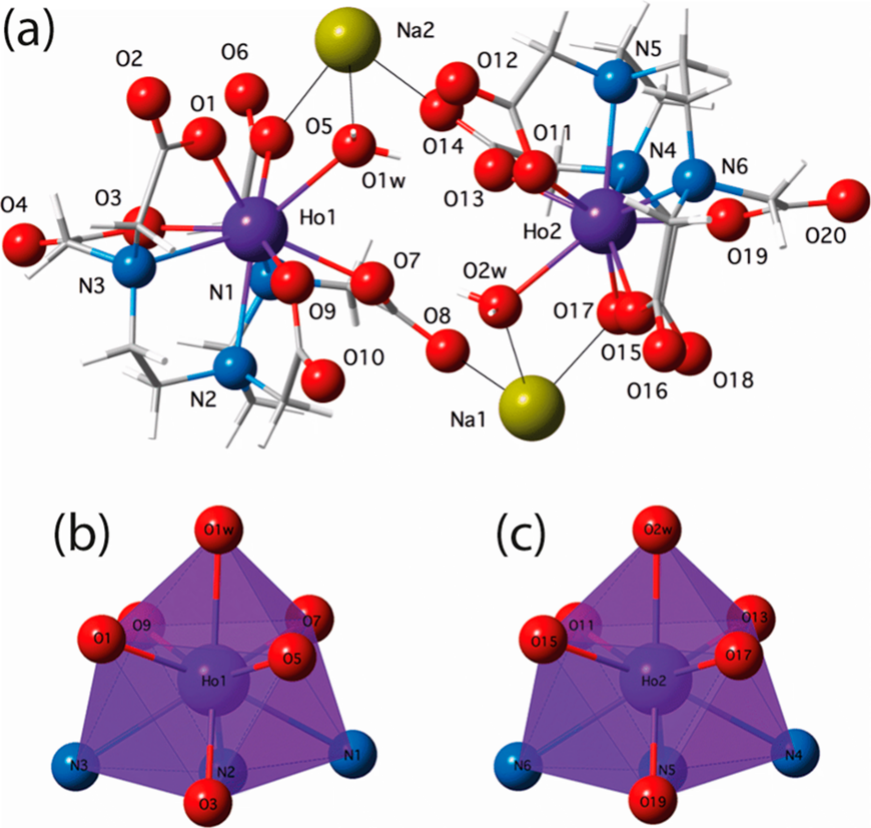
Perspective view of the diholmium(III) entity in **1** (a) showing the double sodium(I)-bridged mononuclear units with the atom numbering scheme. The remaining Na and O atoms from the sodium(I)-coordinated and hydrogen-bonded crystallization water molecules are omitted for clarity. Solid lines represent the Na–O bonds. Views of the metal coordination polyhedra for the two crystallographically independent Ho1 (b) and Ho2 (c) atoms.

The diholmium(III) entities are further connected through the six-coordinate (Na1 and Na2) and five-coordinate (Na3) Na atoms to give a carboxylate/aqua-bridged sodium(I)–holmium(III) layer of mixed square-decagonal topology growing within the crystallographic *ab* plane (Figure S4a). The remaining six-coordinate Na4 atoms act as additional bridges between adjacent heterobimetallic Na^I^Ho^III^ layers to give a pillared-layer three-dimensional (3D) network (Figure S4b), featuring small rectangular pores filled by most of the hydrogen-bonded crystallization water molecules (Figure S5). The Ho1···Ho2 separation (*r*) through the Na1 and Na2 bridges is 6.414 Å, while the shortest intra- and interlayer Ho1···Ho1^I^ and Ho2···Ho2^II^ separations are 8.190 and 10.074 Å (symmetry codes: I, −*x* + ^1^/_2_, −*y* + ^1^/_2_, −*z* + ^1^/_2_; II, −*x*, −*y*, −*z*).

The static magnetic properties of **1** have been investigated by variable-temperature (2–300 K) molar dc magnetic susceptibility (χ_M_) measurements and variable-temperature (2–20 K) and variable-field (0–8 T) molar magnetization (*M*) measurements (see the experimental section). The χ_M_*T* versus *T* and *M* versus *H*/*T* plots (Figure S6) are typical of a highly anisotropic ^5^I_8_ ground state (*J* = 8 with *S* = 2 and *L* = 6) resulting from the large first-order spin–orbit coupling in Ho^III^ ions.^17^

At room temperature, χ_M_*T* for **1** is 14.40 cm^3^ mol^–1^ K. This value is close to that calculated for one Ho^III^ ion (14.07 cm^3^ mol^–1^ K).^15^ Upon cooling, χ_M_*T* continuously decreases to 5.84 cm^3^ mol^–1^ K at 2.0 K (Figure S6a). Ground ^5^I_8_ and first excited ^5^I_7_ states are well-separated (Δ*E* = −8λ with λ_0_ = −541 cm^–1^) so that the former is the only populated below room temperature.^17^ The large deviations of the experimental magnetic susceptibility data from the Curie law are then attributed to the action of the ligand-field (LF) potential on the ^5^I_8_ ground state (see the Computational details).^18,19^ This LF effect causes the splitting of their 17 components into one singlet (*m*_*J*_ = 0) and eight Kramers doublets (*m*_*J*_ from ±1 to ±8), which is ultimately responsible for the high local magnetic anisotropy of the Ho^III^ ion. This conclusion is further supported by the isothermal magnetization curves in the temperature range 2–20 K (Figure S6b). Hence, the *M* value at *T* = 2 K for *H* = 8 T (6.08 Nβ) is well below the calculated value of the magnetization saturation for one Ho^III^ ion (10 Nβ).^17^ Moreover, the isothermal magnetization curves do not superpose and largely deviate from the corresponding Brillouin function for a heptadecet state (solid line in Figure S6b).^17^

The dynamic magnetic properties of **1** have been investigated by variable-temperature (2–7 K) and variable-field (0–1 T) in-phase (χ_M_′) and out-of-phase (χ_M_″) molar ac magnetic susceptibility measurements in the frequency range 1–10 kHz (see the experimental section). The χ_M_′ and χ_M_″ versus *T* and *H* plots reveal a large temperature and field dependence of the spin dynamics (Figures 2 and S7 and S8).

**Figure 2:**
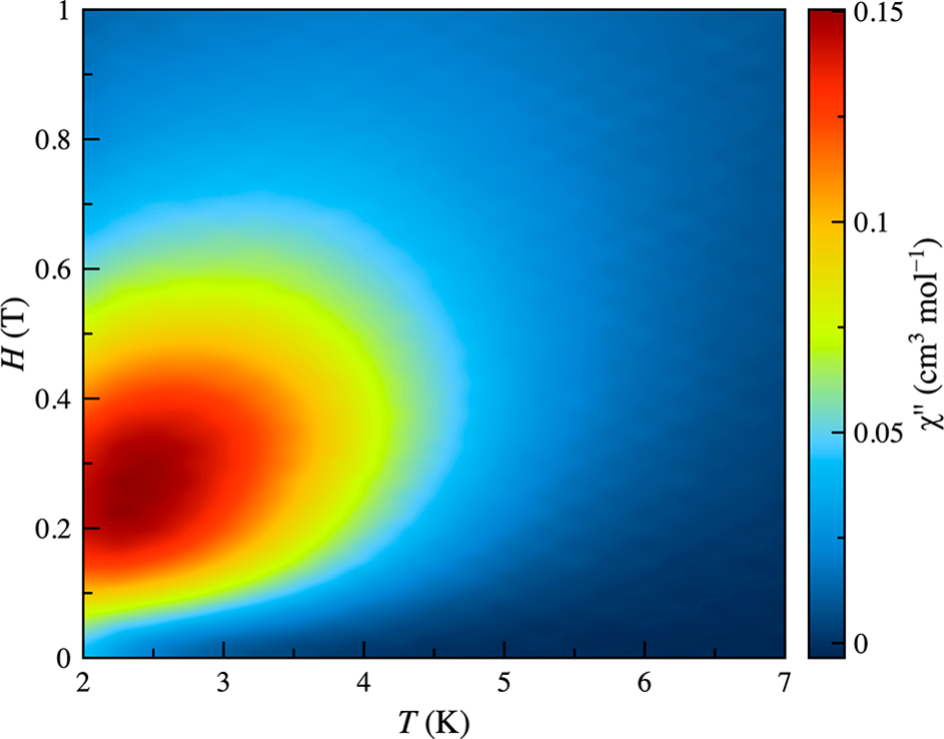
3D contour color map for the temperature and field dependence of χ_M_″ for **1** at 10 kHz of the ±5 Oe oscillating field in the magnetic field and temperature ranges 0–1 T and 2–7 K.

In the absence of a dc magnetic field (*H* = 0), there is an incipient χ_M_″ signal with no χ_M_′ maximum at 10 kHz (Figure S7). However, when a relatively small dc magnetic field is applied, χ_M_″ shows maxima in the temperature range 2–4 K, with 0.25 T being the optimal working magnetic field (Figure 2). Likewise, a maximum appears in the χ_M_″ versus *H* plots that broadens below 4 K (Figure S8b), showing the appearance of a second competing relaxation process that is clearly noticeable only at 2 K (*H* ≈ 0.2 T). These field-induced slow magnetic relaxation (SMR) effects are typical of Ln SIMs, as was earlier reported for the related complexes of the formulas Na[Gd^III^(EDTA)(H_2_O)_3_]·5H_2_O and Na[Dy^III^(DOTA)(H_2_O)]·4H_2_O (Scheme S1).^20−22^

To confirm the occurrence of field-induced SIM behavior, the influence of the frequency on the spin dynamics was then investigated at the optimum applied dc magnetic field. The χ_M_′ and χ_M_″ versus *T* and ν plots in the frequency range 1–10 kHz at *H* = 0.25 T are compared in Figures S9 and S10. The magnetic relaxation time (τ) values for **1** in the temperature range 2.25–4 K at *H* = 0.25 T can be calculated from a joint analysis of the χ_M_ and χ_M_″ versus ν plots through the generalized Debye equations, where α describes the distribution of the magnetic relaxation times (solid lines in Figures S9 and S10). The moderate α values in the range 0.36–0.44 support a wide distribution of magnetic relaxation processes (0 and 1 for single and infinite magnetic relaxation processes).

The estimated τ values at *H* = 0.25 T are represented in the form of the ln τ versus 1/*T* (so-called Arrhenius) plots in Figure S11. The experimental data follow a linear Arrhenius law characteristic of a single thermally activated magnetic relaxation process that was satisfactorily simulated through a two-phonon Orbach mechanism [τ^–1^ = τ_0_^–1^ exp(−*U*_eff_/*k*_B_*T*)]. The ln τ versus ln *T* plot does not show any temperature region in which direct relaxation prevails (data not shown). The values of τ_0_ and *U*_eff_ [3.2(4) × 10^–8^ s and 6.8(2) cm^–1^] are within the wide range found for the other few examples of Ho SIMs (Table S3).^23−31^

The magnetothermal properties of **1** have been investigated by variable-temperature (2–20 K) and variable-field (0–8 T) magnetization measurements (Figure S12). The resulting magnetic entropy change (−Δ*S*_M_) versus *T* and Δ*H* plots obtained through a numerical approach described elsewhere according to the Maxwell equation [Δ*S*_M_ = ∫(∂*M*/∂*T*) d*H*] are depicted in Figures 3 and S13.^32^

**Figure 3:**
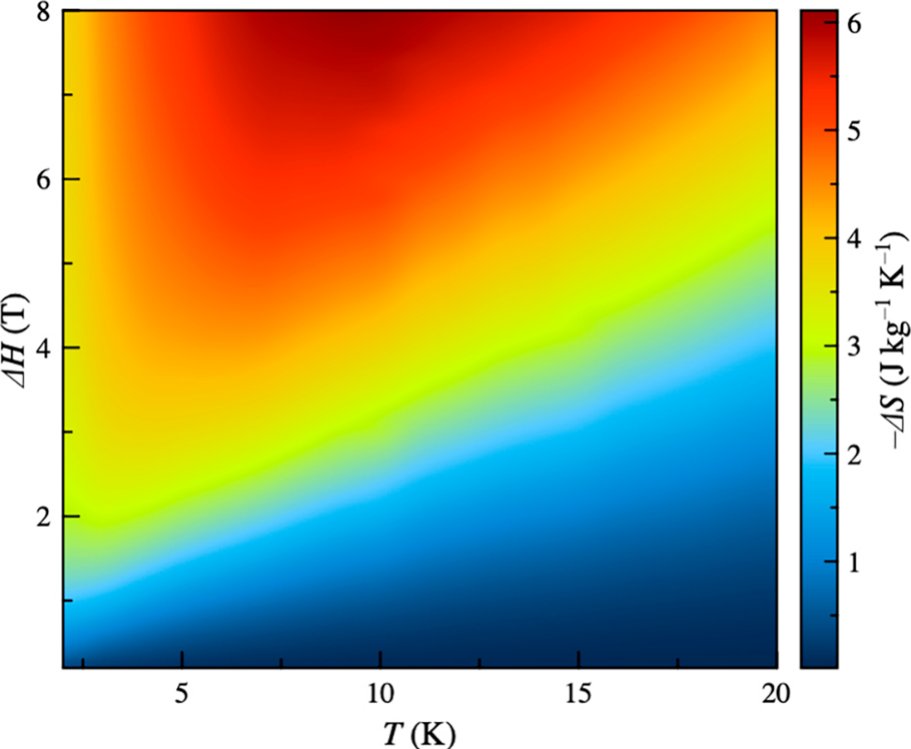
Contour color map for the temperature and field dependence of the −Δ*S*_M_ for **1** in the field and temperature ranges 0–8 T and 2–20 K.

The contour map in Figure 3 indicates that the optimal working temperature is 9.5 K, reaching a CMR performance higher than 75% for Δ*H* > 4 T or 50% at 2 K for Δ*H* = 2 T because of the rapid saturation of the magnetic entropy. Indeed, high magnetic entropy global changes and large slopes of the isothermal magnetic entropy curves are mandatory for potential applications as molecular cryomagnetic coolants.

Blockage of the magnetization at low temperatures under a weak dc magnetic field (*T*_B_ < 2.5 K for *H* = 0.25 T) should preclude the observation of large magnetocaloric effects (MCEs), as suggested by the lack of a −Δ*S*_M_ maximum for Δ*H* < 1 T. The increase of the magnetic field induces a progressive shift of this maximum covering a wide temperature range above the liquid-helium temperature, from 3.2 (Δ*H* = 2 T) to 9.5 K (Δ*H* = 8 T) (Figure S13a). This feature is also related to the MCE efficiency and eventually to the practical applications of **1** as a cryomagnetic coolant in the low temperature range. By comparison, the mononuclear gadolinium(III) polyoxometalates of the formulas Na_9_[Gd(W_5_O_18_)_2_] and K_12_[Gd(P_5_W_30_O_110_)(H_2_O)], abbreviated as GdW_10_ and GdW_30_, exhibit SMR effects at very low temperatures (*T*_B_ < 0.1 K) in the absence of a magnetic field, with −Δ*S*_M_ maxima just below the liquid-helium temperature.^8,9^

The magnetic entropy increases with the magnetic field, and it tends asymptotically to a thermally dependent value reached more quickly at lower temperatures (Figure S13b). Hence, the maximum −Δ*S*_M_ value in gravimetric units for Δ*H* = 8 T increases from 4.5 (*T* = 20 K) up to 6.2 J kg^–1^ K^–1^ (*T* = 10 K), and then it further decreases to 3.8 J kg^–1^ K^–1^ (*T* = 2 K). These values are higher than those found for GdW_10_ and GdW_30_ (−Δ*S*_M_ = 1.9 and 4.7 J kg^–1^ K^–1^ at *T* = 1.3 and 1.8 K, respectively, for Δ*H* = 7 T),^9^ comparing well with those of metallic holmium and related intermetallic alloys (HoB_2_ and HoAl_2_), recently proposed as cryomagnetic coolants just above the hydrogen liquefaction temperature.^33−35^

Yet the maximum −Δ*S*_M_ value in molar units for **1** (−Δ*S*_M_ = 4.7 J mol^–1^ K^–1^ at *T* = 10 K for Δ*H* = 8 T) is rather lower than the limiting value for one Ho^III^ ion with no zero-field splitting (zfs) [Δ*S*_M_ = *R* ln(2*J* + 1) = 23.6 J mol^–1^ K^–1^ with *J* = 8]. Although the Ho^III^ ion possesses the highest total angular momentum along the lanthanide series, this almost 5-fold reduction of the MCE efficiency is expected because of its rather high magnetic anisotropy, as discussed above. In fact, the large zfs of the ground heptadecet state strongly minimizes the magnetic entropy at zero field, thereby reducing the maximal magnetic entropy change (in absolute value) under the application of a magnetic field.

In summary, **1** behaves as a novel Ln SIM with a significant field dependence of the SMR at relatively low blocking temperatures. Likewise, it exhibits moderate MCE in a wide temperature range just above the occurrence of the field-induced magnetization blockage, with strong field-dependent magnetic entropy maxima between the liquid helium and hydrogen temperatures. Despite their limited CMR performance, the results reported herein illustrate the potential of magnetically anisotropic holmium(III)-based SIMs as prototypes of molecular cryomagnetic coolants operating near the strategically relevant hydrogen liquefaction temperature.

## Supporting Information Available

Synthetic, structural, magnetic, and magnetocaloric data (Tables S1–S3, Schemes S1 and S2, and Figures S1–S13) (PDF)

## Accession Codes

CCDC 2069070 contains the supplementary crystallographic data for this paper. These data can be obtained free of charge via www.ccdc.cam.ac.uk/data_request/cif, or by emailing data_request@ccdc.cam.ac.uk, or by contacting The Cambridge Crystallographic Data Centre, 12 Union Road, Cambridge CB2 1EZ, UK; fax: +44 1223 336033.
