# Supplementary material for: Holmium(III) Single-Ion Magnet for Cryomagnetic Refrigeration Based on an MRI Contrast Agent Derivative
Source: Inorg Chem. 2021 Aug 23;60(17):12719–23. doi: 10.1021/acs.inorgchem.1c01905 (PMC8424628; doi:10.1021/acs.inorgchem.1c01905)
Supplement: Supplementary file 1 — ic1c01905_si_002.pdf [file ic1c01905_si_002.pdf]

Supplementary Information (SI) for the manuscript:

Holmium(III) Single-Ion Magnet for Cryomagnetic Refrigeration Based on an MRI Contrast Agent Derivative

Borja Rodríguez-Barea, Júlia Mayans, Renato Rabelo, Adrián Sanchis-Perucho, Nicolás Moliner, José Martínez-Lillo,* Miguel Julve, Francesc Lloret, Rafael Ruiz-García and Joan Cano*

Instituto de Ciencia Molecular (ICMol)/Departament de Química Inorgànica, Facultat de Química, Universitat de València, 46980 Paterna (València), Spain

Materials

Diethylenetriamine-*N,N,N',N'',N''*-pentaacetic acid (H_5DTPA), sodium(I) bicarbonate, and holmium(III) oxide were of reagent grade; they were purchased from commercial sources and used as received.

Preparation and general physicochemical characterization

$Na_2[Ho^{III}(DTPA)(H_2O)]\cdot 8H_2O$ (**1**): Powdered Ho_2O_3 (0.95 g, 2.5 mmol) was added in three small portions to a hot aqueous solution (50 mL) of H_5DTPA (1.97 g, 5.0 mmol) under stirring. The mixture was heated under reflux for *ca.* 5 h until the solution became transparent (pH = 1.0). The pH of the solution was adjusted to 6.5 by dropwise addition of an aqueous solution of $NaHCO_3$ (0.84 g, 10.0 mmol). The resulting pink solution was concentrated to 25 mL and then filtered on paper. After several weeks of slow evaporation of the filtered solution, an oil was formed and then slowly transformed into a crystalline solid by standing at room temperature under atmospheric conditions after several days (Figure S1). A small amount of X-ray quality light pink prims of **1** were grown. The chemical identity and purity of the bulk crystalline material were further confirmed by X-ray powder diffraction (XRPD) (Figure S2), whose pattern is identical to the calculated one from the single-crystal XRD analysis. It shows a whitish pink color under white light illumination, while a weak purple luminescence, typical of holmium(III) complexes, appears under UV-A light irradiation at 365 nm (Figure S3). Yield: 3.4 g (90%). Anal. Calcd: C, 22.09; H, 4.77; N, 5.52%. Found: C, 21.65; H, 4.53; N, 5.36%. IR (KBr, cm^{-1}): 3472m [ν(OH) from H_2O], 3102w, 3082w, 2940w [ν(CH) from DTPA], 1604s [$\nu_{as}(CO_2)$ from DTPA], 1404s [$\nu_s(CO_2)$ from DTPA].

Physical techniques

Elemental (C, H, N) analyses were performed by the Servei Central de Suport a la Investigació Experimental (SCSIE) de la Universitat de València. Fourier-transform infrared (FTIR) spectra were recorded on a Nicolet-5700 spectrophotometer as KBr pellets. Powder X-ray diffraction (XRD) patterns of powdered crystalline samples were collected at room temperature on a D8 Advance A25 Bruker diffractometer by using graphite-monochromated Cu-Kα radiation (λ = 1.54056 Å). Optical microscopy images were carried out at 80 amplification using a Nikon SMZ1000 optical microscope equipped with a Nikon Digital Sight DS-Fi1 camera. Images were taken with the NIS-Elements D software under illumination with a UV-A (λ_{exc} = 365 nm) lamp.

Magnetic measurements

Variable-temperature (2–300 K) direct current (*dc*) magnetic susceptibility measurements under applied magnetic field of 0.5 T and variable-temperature (2–20 K) and variable-field (0–8 T) magnetization measurements on powdered crystalline samples were carried out using Quantum Design Superconducting Quantum Interference Devices (SQUID) magnetometer and Physical Property Measurement System (PPMS). The samples were embedded in *n*-eicosane to prevent any crystal reorientation.

Variable-temperature (2–7 K) and variable-field (0–1 T) alternating current (*ac*) magnetic susceptibility measurements under ±5.0 Oe oscillating field at frequencies in the range 1–10 kHz were performed with a Quantum Design PPMS. The magnetic susceptibility data were corrected for the diamagnetism of the constituent atoms and the sample holder.

Computational details

The magnetic susceptibility data of **1** were simulated from a ligand field (LF) model. According to Steven's notation and the molecular D_{3h} pseudo-symmetry,[1,2] the appropriate LF Hamiltonian for **1** would be expressed by equation (1), where $\hat{O}_k^q$ stand for the operator equivalents containing the simplest $\hat{J}^2$, $\hat{J}_z$, $\hat{J}_+$, and $\hat{J}_-$ ones, while $A_k^q\langle r^k\rangle$ are the coefficients of the LF parameters.

$$\hat{H}_{LF} = A_2^0\langle r^2\rangle\hat{O}_2^0 + A_4^0\langle r^4\rangle\hat{O}_4^0 + A_6^0\langle r^6\rangle\hat{O}_6^0 + A_6^6\langle r^6\rangle\hat{O}_6^6 \quad (1)$$

The application of this Hamiltonian on the $^5I_8$ ground state allowed the simulation of the experimental $\chi_M T$ *vs.* T data (Figure S6a) with the following best-fit parameters: $A_2^0\langle r^2\rangle = 284.1$, $A_4^0\langle r^4\rangle = 758.6$, $A_6^0\langle r^6\rangle = 2.8$, and $A_6^6\langle r^6\rangle = 5.0$, which are of the same order of magnitude than those in the literature.[19] A temperature-independent paramagnetism of 1590×10^{-6} cm^3 mol^{-1} was considered. The agreement factor defined as $F = \sum \frac{(P_{exp} - P_{calc})^2}{P_{exp}^2}$ was 5.8×10^{-5}. However, several sets of values can be found that correctly simulate the experimental data, even without the last two terms of the Hamiltonian and particularly the last one.

X-ray crystallographic data collection and structure refinement

Single-crystal X-ray diffraction data of **1** were collected on a Bruker D8 Venture diffractometer with a PHOTON II detector using monochromatic Mo-K$_\alpha$ radiation (λ = 0.71073 Å). The structure was solved by standard direct methods and subsequently completed by Fourier recycling by using the SHELXTL software package. The obtained

[1] Rudowicz, C.; Karbowiak, M. Disentagling Intrigate Web of Interrrelated Notions at the Interface between the Physical (Crystal Field) Hamiltonians and the Effective (Spin) Hamiltonians. *Coord. Chem. Rev.* **2015**, *287*, 28–63.
[2] Layfield, R. A.; Murugesu M. (Eds.), *Lanthanides and Actinides in Molecular Magnetism*; Wiley-VCH Publishers: New York, 2015.

models were refined with 2017/1 version of SHELXL against F^2 on all data by full-matrix least squares.[3] All non-hydrogen atoms were refined anisotropically. The hydrogen atoms of the water molecules were located on the ΔF map and refined with restraints. The graphical manipulations and calculations were performed with the CrystalMaker and Mercury programs.[4,5] Crystallographic data (excluding structure factors) for the structure reported in this work have been deposited with the Cambridge Crystallographic Data Centre as supplementary publication number CCDC–2069070. Copies of the data can be obtained free of charge on application to CCDC, 12 Union Road, Cambridge CB21EZ, UK (fax: (+44) 1223−336−033; e-mail: deposit@ccdc.cam.ac.uk).

[3] *SHELXTL-2017/1*, Bruker Analytical X-ray Instruments; Bruker: Madison, WI, USA, 2017.
[4] *CrystalMaker*, CrystalMaker Software, Bicester, England, 2021.
[5] *Mercury*, The Cambridge Crystallographic Data Centre, Cambridge, UK, 2020.

Table S1. Summary of Crystallographic Data and Structure Refinement for 1

Formula	$C_{28}H_{72}Ho_2N_6Na_4O_{38}$
M (g mol^{-1})	1522.73
Crystal system	Monoclinic
Space group	$P2_1/n$
a (Å)	18.0903(7)
b (Å)	14.9403(6)
c (Å)	20.6925(8)
α (°)	90
β (°)	108.302(2)
γ (°)	90
V (Å^3)	5309.7(4)
Z	4
ρ_{calc} (g cm^{-3})	1.905
μ (mm^{-1})	3.102
T (K)	120(2)
Reflect. collcd.	62357
Reflect. obs. [$I > 2\sigma(I)$]	7360
Data/Restraints/Parameters	9370/60/846
$R_1{}^a$ [$I > 2\sigma(I)$]	0.0334
$wR_2{}^b$ [$I > 2\sigma(I)$]	0.0592
S^c	1.078

$^aR_1 = \sum(|F_o| - |F_c|)/\sum|F_o|$. $^bwR_2 = [\sum w(F_o^2 - F_c^2)^2/\sum w(F_o^2)^2]^{1/2}$. $^cS = [\sum w(|F_o| - |F_c|)^2/(N_o - N_c)]^{1/2}$.

Table S2. Selected Structural Data for 1[a]

	Ho1	Ho2
Ho–O_w[b] (Å)	2.506(4)	2.468(4)
Ho–O[c] (Å)	2.349(3)	2.360(3)
Ho–N_c[d] (Å)	2.552(4)	2.549(4)
Ho–N_t[e] (Å)	2.652(4)	2.639(4)
N_t–Ho–N_t[f] (°)	119.53(13)	119.33(13)
N_t–Ho–O_w[f] (°)	115.82(13)	117.34(13)
	124.61(13)	123.30(13)
Δ[g] (Å)	0.030	0.026
δ[h] (°)	173.83	173.72
τ[i] (°)	4.27	4.22
v/h[j]	1.105	1.117

[a]The structural data correspond to each crystallographically independent holmium atom. [b]Value of the metal bond length with the oxygen atom from the water molecule. [c]Average value of the metal bond length with the carboxylate oxygen atoms from the DTPA ligand. [d]Value of the metal bond length with the central amine nitrogen atoms from the DTPA ligand. [e]Average value of the metal bond lengths with the terminal amine nitrogen atoms from the DTPA ligand. [f]Value of the metal interbond angles subtended by the three capping donor atoms of the tricapped trigonal prism (TCTPR). [g]Value of the metal deviation from the mean plane formed by the three capping donor atoms of the TCTPR. [h]Value of the dihedral angle between the two opposite triangular prismatic faces of the TCTPR. [i]Value of the trigonal twist angle between the two opposite triangular prismatic faces of the TCTPR. [j]Value of the elongation parameter of the rectangular prismatic faces of the TCTPR.

Table S3. Selected ac Magnetic Data for 1 and literature reported Ho^{III} SIMs

Complex[a]	H^b (T)	$\tau_0{}^c$ (s)	$U_{eff}{}^c$ (cm^{-1})	$T_B{}^d$ (K)	Ref.
$n$$Bu_4N[Ho(Pc)_2]$	0	n.a.	n.a.	0.5	23
$K_{12}[HoP_5W_{30}O_{110}]$	0	6.0×10^{-3}	0.6	2.0	24
$K_9[Ho(W_5O_{18})_2]$	0	n.a.	n.a.		25
$K_{13}[Ho(\beta_2\text{-}SiW_{11}O_{39})_2]$	0	n.a.	n.a.		25
$HoSc_2N@C_{80}$	0.2	1.7×10^{-5}	11.5		26
$[Ho(CyPh_2PO)_2(H_2O)_5]I_3$	0	1.7×10^{-11}	237	1.0	27
$[Ho(OC_6H_3Me_2)_2(py)_5]BPh_4$	0	1.1×10^{-11}	278		28
$[Ho(OCHMePh)_2(py)_5]BPh_4$	0	0.7×10^{-11}	349		28
$[Ho(OSiMe_3)_2(py)_5]BPh_4$	0	1.3×10^{-12}	500		28
$Ho(CH_2SiMe_3)_3(THF)_2$	0	1.7×10^{-10}	53		29
$[Ho(HMPA)_2(H_2O)_5]Cl_3$	0	0.8×10^{-10}	203		30
$[Ho(HMPA)_2(H_2O)_5]Br_3$	0	1.4×10^{-11}	224		30
$Ho(Opy)_4(H_2O)_2Co(CN)_6$	0	3.3×10^{-8}	89.8		31
$Ho(Opy)_4(H_2O)_2Rh(CN)_6$	0	4.0×10^{-8}	86.7		31
$Ho(Opy)_4(H_2O)_2Ir(CN)_6$	0	4.8×10^{-6}	78.7		31
**1**	0.25	3.2×10^{-8}	6.8		This work

[a]Ligand abbreviations: Pc = phthalocyaninate; $CyPh_2PO$ = cyclohexyldiphenylphosphine oxide; py = pyridine; $HOSiMe_3$ = trimethylsilanol; HOCHMePh = 1-phenylethanol; $HOC_6H_3Me_2$ = 3,5-dimethylphenol; CH_3SiMe_3 = trimethylsilylmethane; THF = tetrahydrofurane; HMPA = hexamethylphosphoric triamide; Opy = 4-pyridone. [b]Value of the applied *dc* magnetic field. [c]Values of the corresponding pre-exponential factor and the effective energy barrier in the Arrhenius law [$\tau^{-1} = \tau_0{}^{-1} \exp(-U_{eff}/k_BT)$]. [d]Value of the blocking temperature for magnetization reversal.

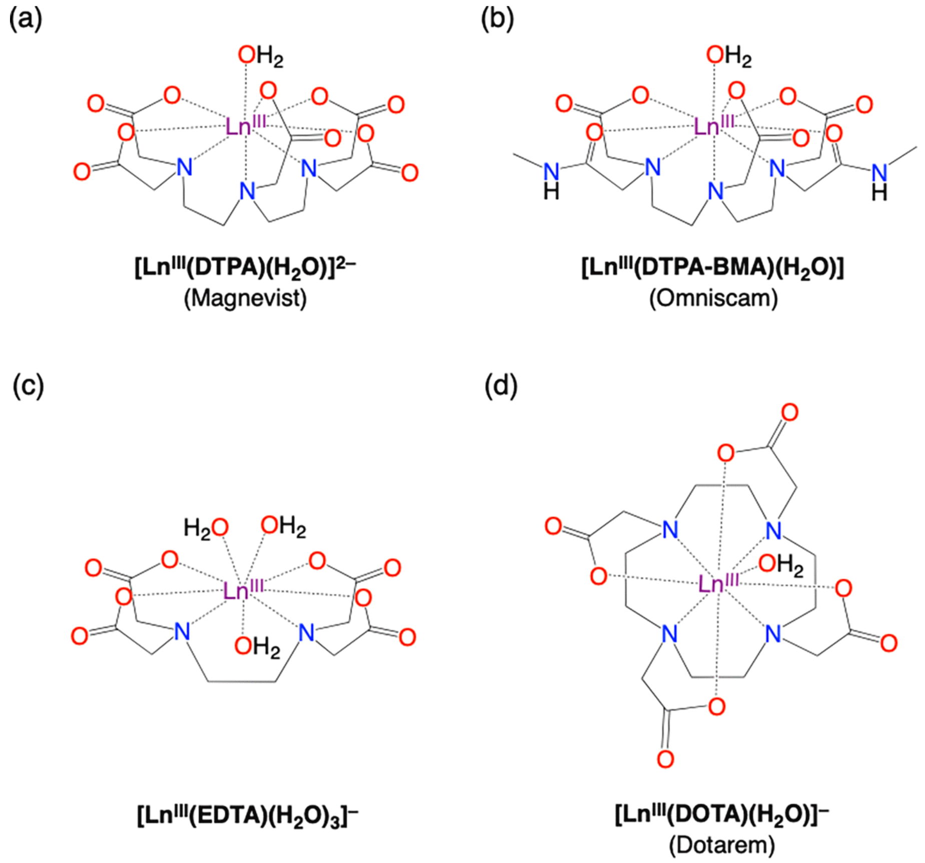

Scheme S1 General chemical structures of mononuclear lanthanide(III)-aqua complexes with either linear or cyclic polyaminocarboxylate ligands and their methylamide derivatives [DTPA = diethylenetriamine-*N,N,N′,N″,N″*-pentaacetate; DTPA-BMA = diethylenetriamine-*N,N′,N″*-tris(acetate)-*N,N″*-bis(methylacetamide); EDTA = ethylenediamine-*N,N,N′,N′*-tetraacetate; DOTA= 1,4,7,10-tetraazacyclododecane-*N,N′,N″,N‴*-tetraacetate]. The commercial names of the parent gadolinium(III) complexes used as MRI contrast agents are given in parentheses.

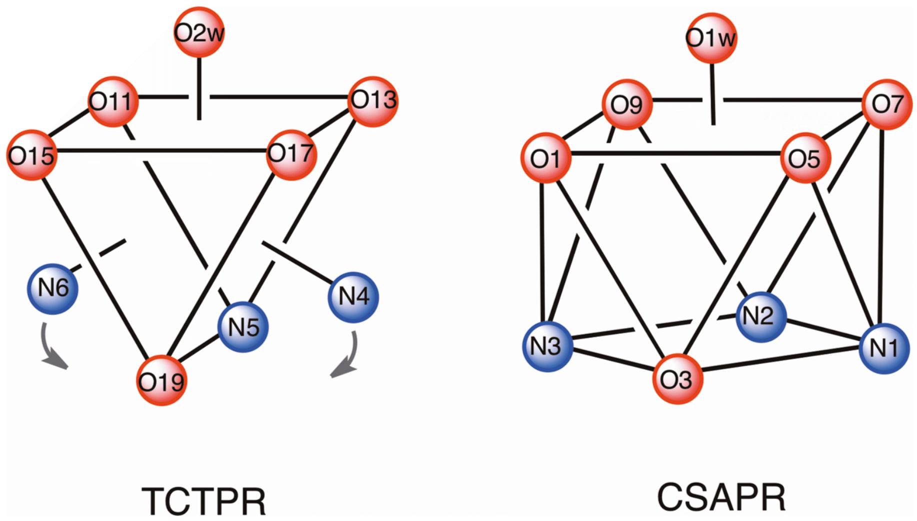

Scheme S2 Drawings of the ideal TCTPR and CSAPR polyhedral for the two crystallographically independent Ho2 and Ho1 atoms with the atom numbering scheme. Gray arrows represent the simple pathway for the structural transformation between each other.

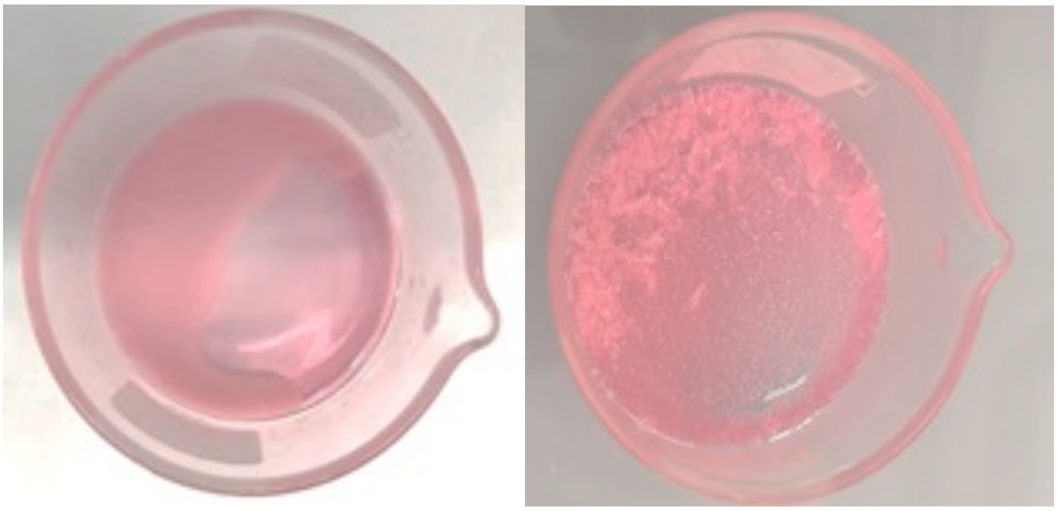

Figure S1. Pictures showing the conversion from an oil (left) to a crystalline solid (right) for **1** after several days on standing under ambient conditions at room temperature.

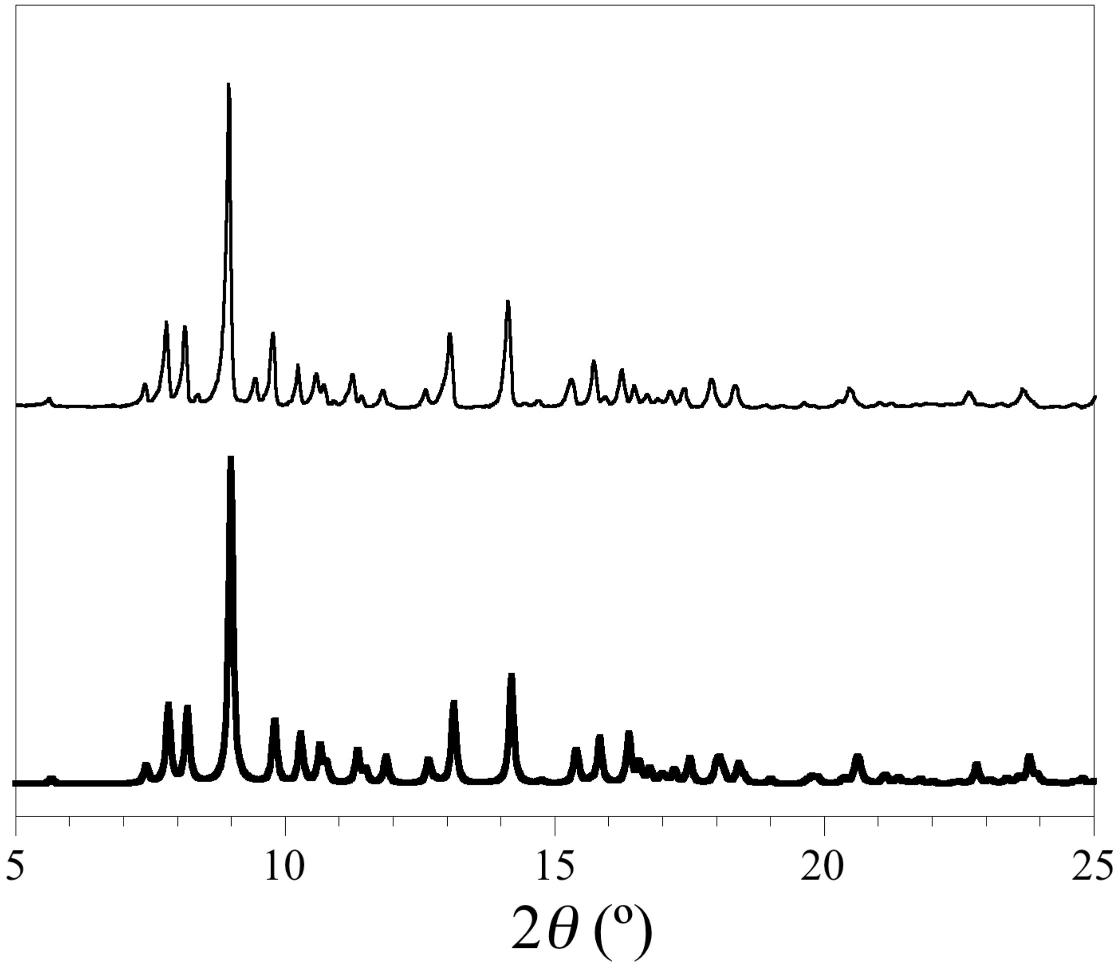

Figure S2. Powder X-ray diffraction (XRD) pattern of **1** (top) compared to the calculated one (bottom).

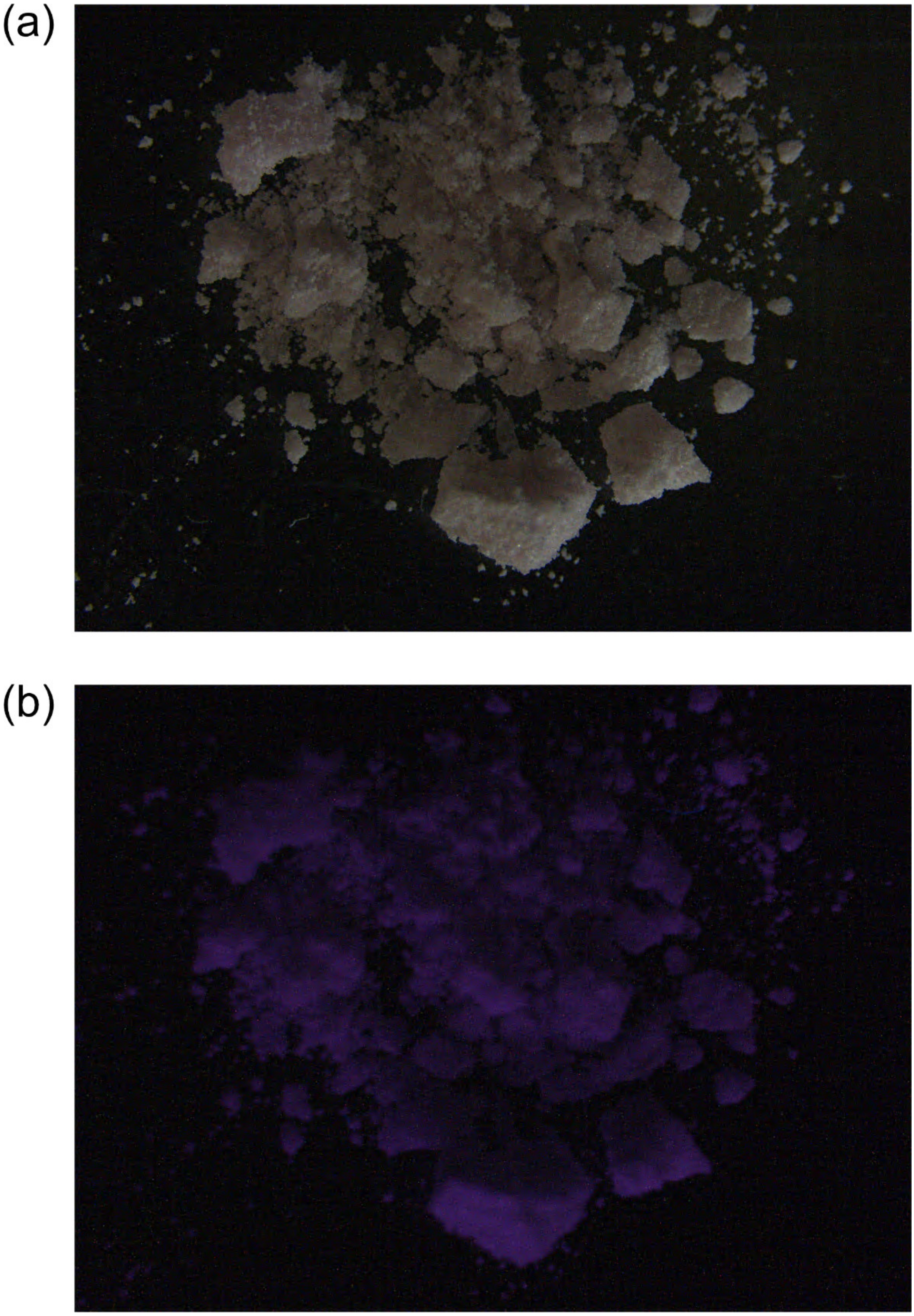

Figure S3. Optical microscopy images of **1** under white light (a) and UV-A lamp (b).

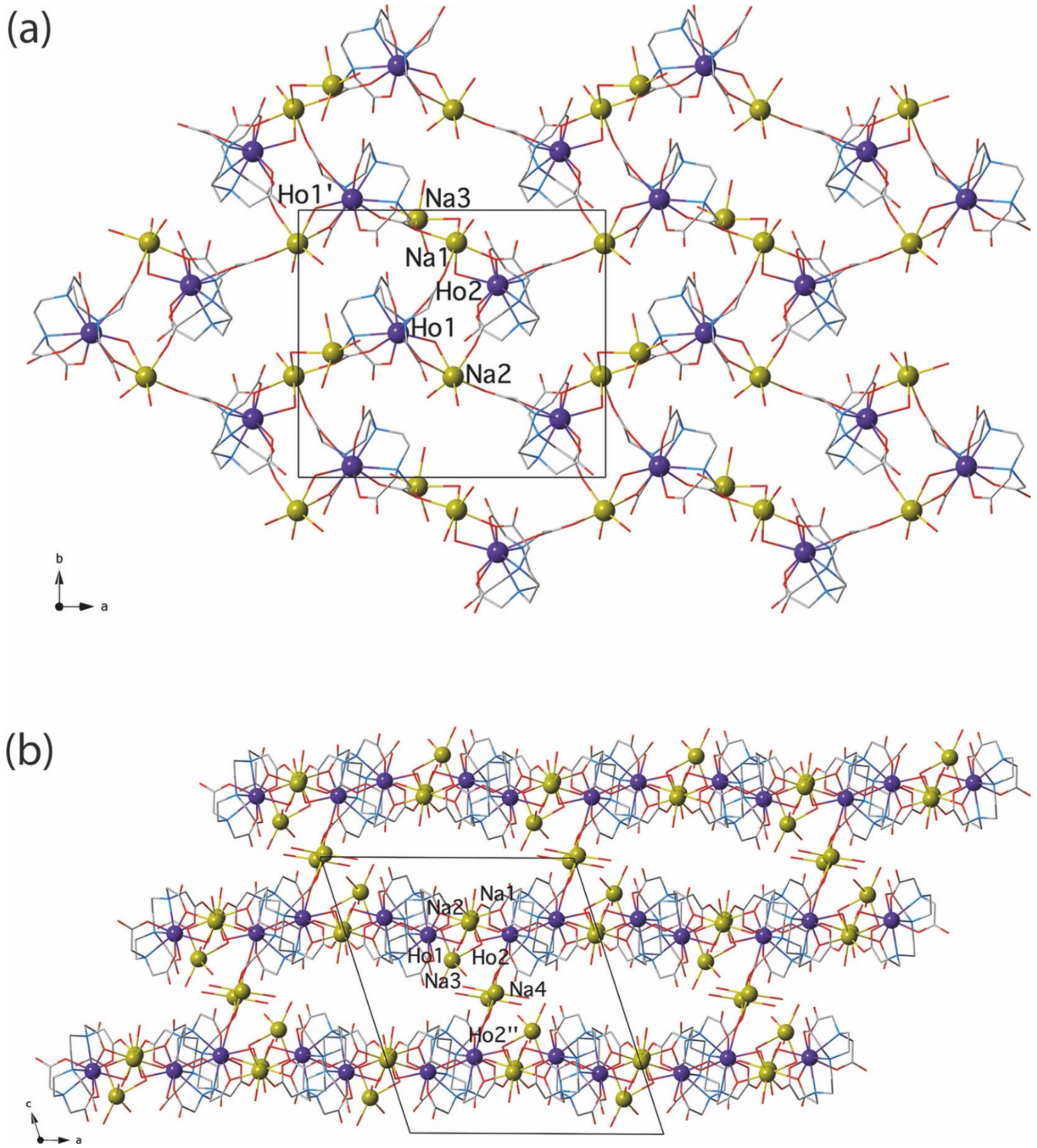

Figure S4. Projection views of a fragment of the mixed square/decagonal carboxylate/aqua-bridged sodium(I)-holmium(III) layer and the pillared layer three-dimensional network of **1** along the crystallographic *c* (a) and *b* axes (b) [Symmetry operations: (I) = $-x + 1/2$, $-y + 1/2$, $-z + 1/2$; (II) = $-x$, $-y$, $-z$]. The crystallization water molecules are omitted for clarity.

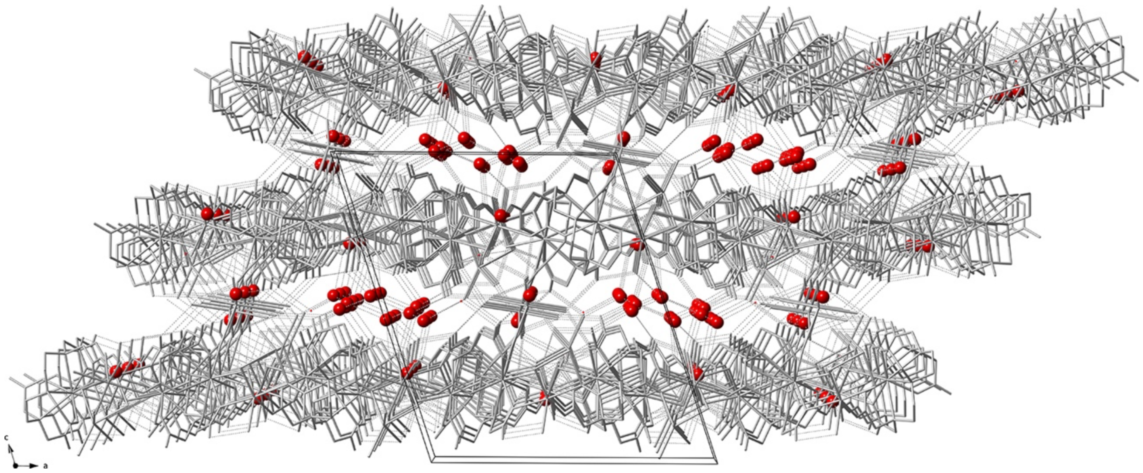

Figure S5. Perspective view of the crystal packing of **1** along the crystallographic *b* axis, showing tiny rectangular pores within the interlayer space. Red spheres represent crystallization water molecules and dotted lines the hydrogen bonds involving them.

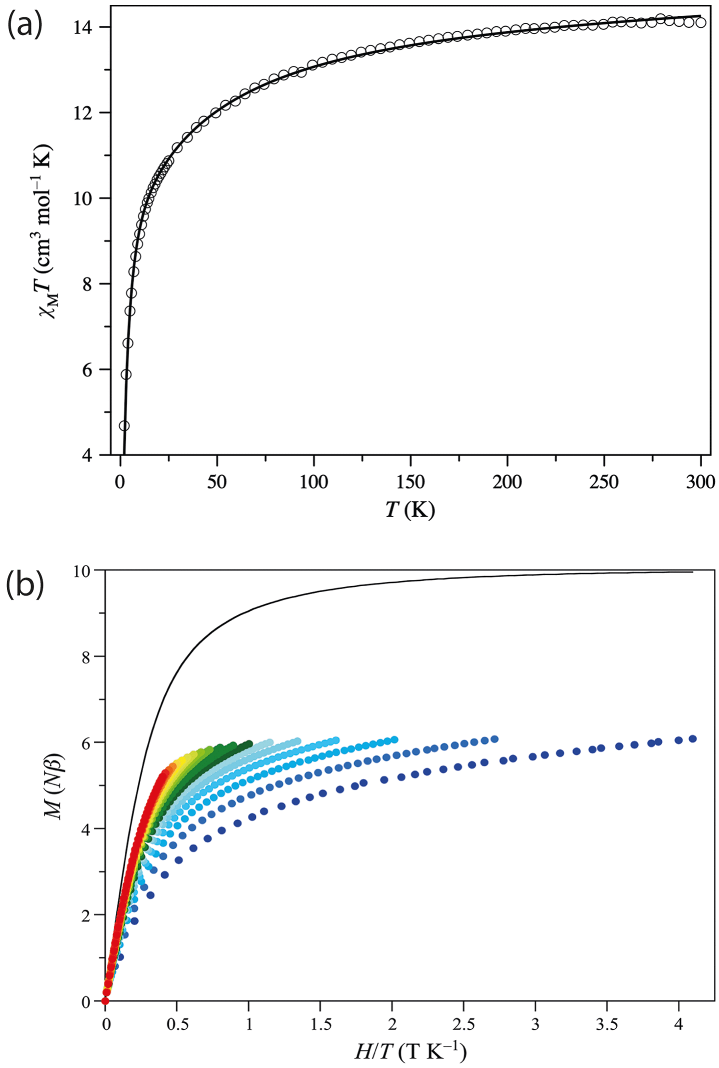

Figure S6. (a) Experimental data (○) and simulated curve (solid line) of the temperature dependence of $\chi_M T$ for **1** under an applied *dc* magnetic field of 0.5 T. (b) Experimental data (●) and simulated Brillouin curve (solid line) of the normalized field dependence of M for **1** in the temperature range 2–20 K (from dark blue to red in steps of 1 K).

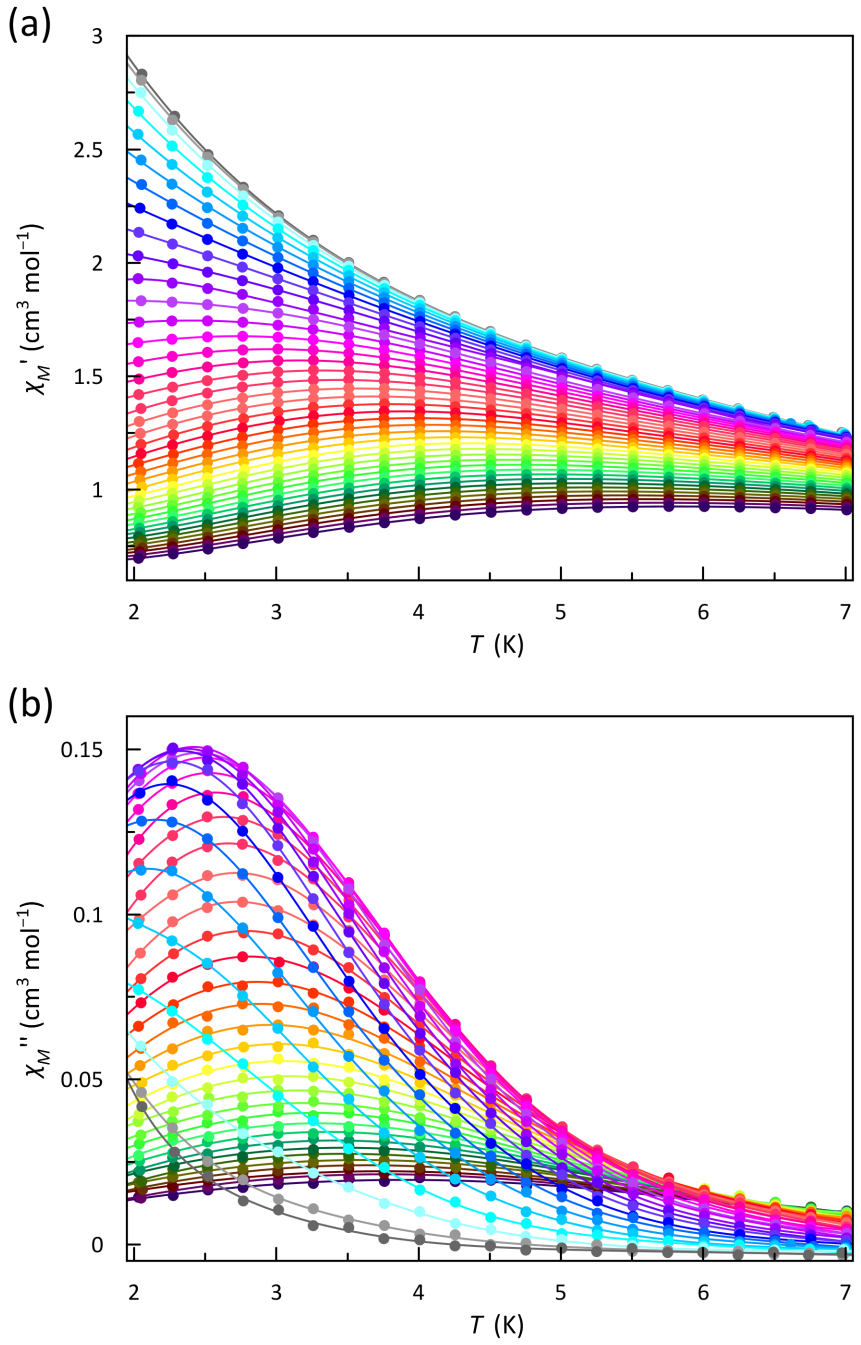

Figure S7. (a) and (b) Temperature dependence of χ_M' and χ_M'' molar *ac* magnetic susceptibilities for **1** at 10 kHz of the ±5 Oe oscillating field in the applied *dc* magnetic field range 0–1 T (from dark grey to dark purple in steps of 0.025 T). The solid lines are only eye-guides.

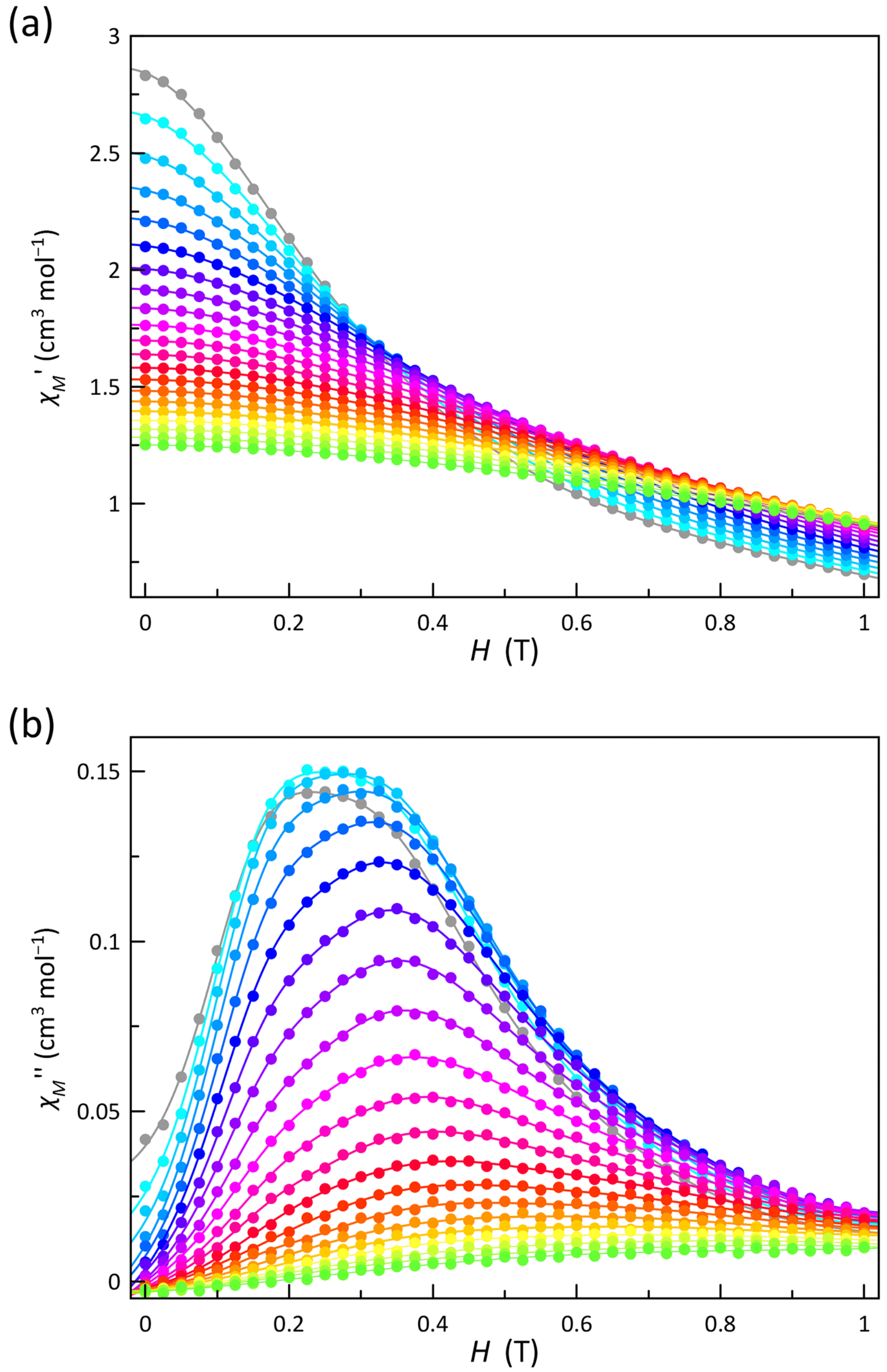

Figure S8. (a) and (b) Field dependence of χ_M' and χ_M'' for **1** at 10 kHz of the ±5 Oe oscillating field in the temperature range 2–7 K (from grey to green in steps of 0.25 K). The solid lines are only eye-guides.

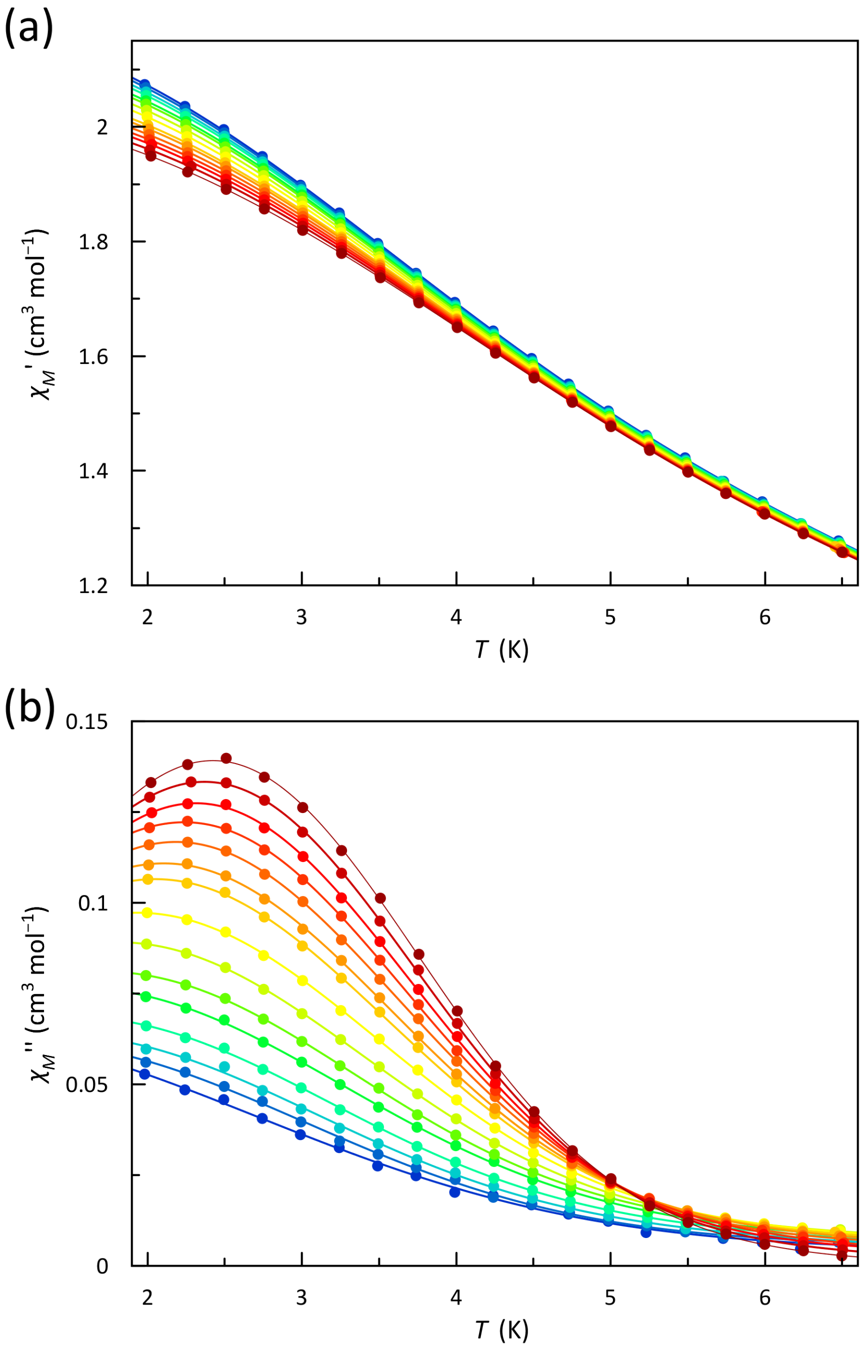

Figure S9. (a) and (b) Temperature dependence of χ_M' and χ_M'' for **1** in the frequency range 1–10 kHz (from dark blue to dark red) of the ±5 Oe oscillating field under an applied *dc* magnetic field of 0.25 T. The solid lines are only eye-guides.

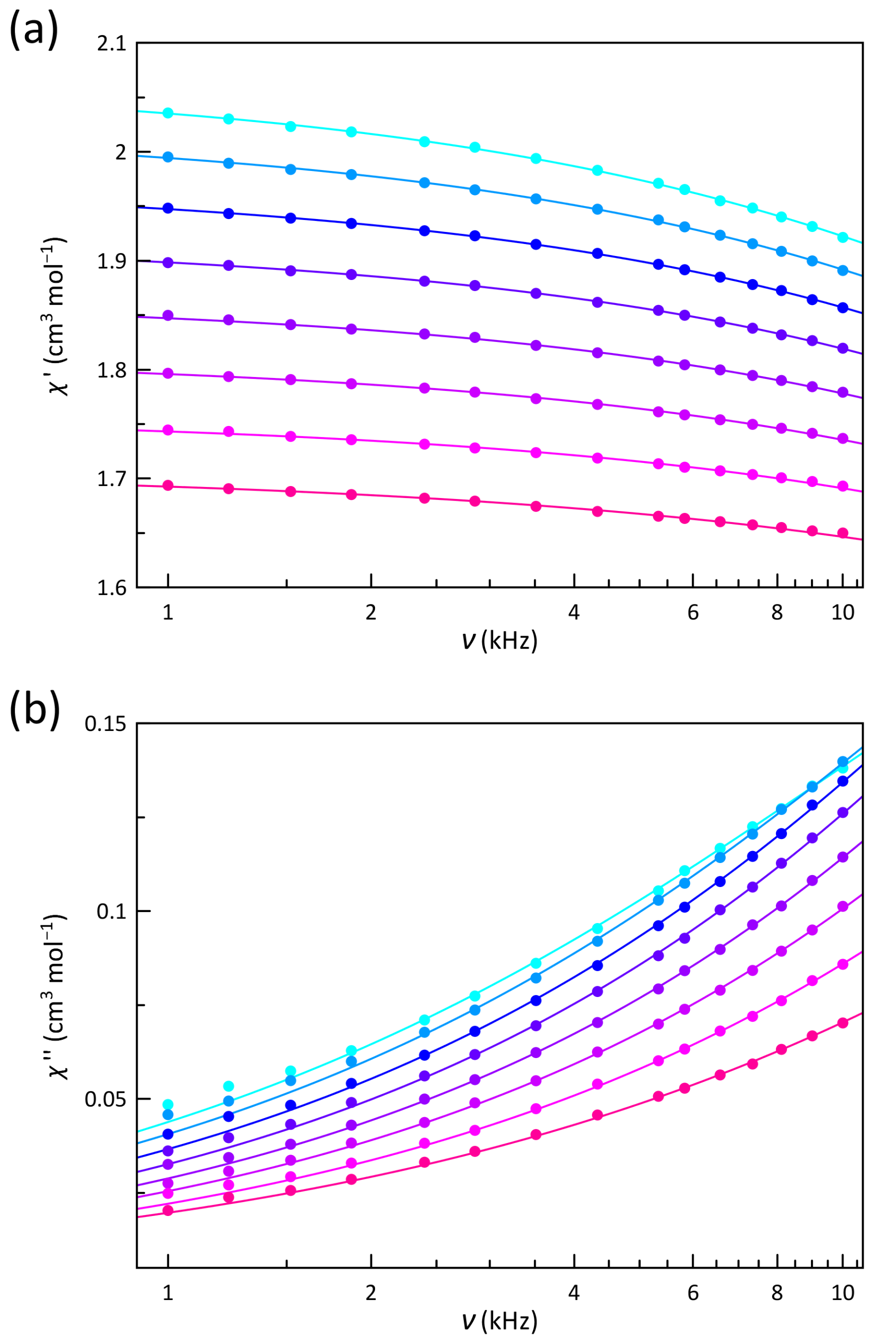

Figure S10. (a) and (b) Frequency dependence of χ_M' and χ_M'' for **1** in the temperature range 2.25–4.0 K (from cyan to red in steps of 0.25 K) under an applied *dc* magnetic field of 0.25 T. The solid lines are the best-fit curves (see text).

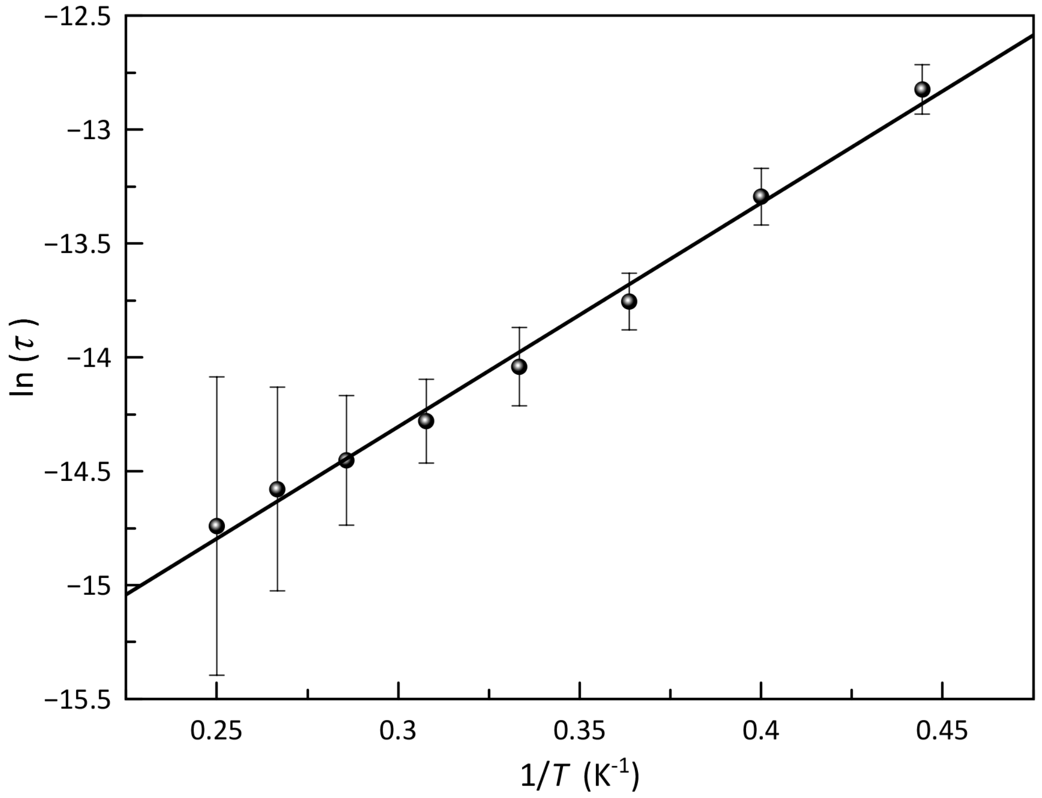

Figure S11. Arrhenius plot of τ for **1** under an applied *dc* magnetic field of 0.25 T. The solid line is the best-fit curve (see text).

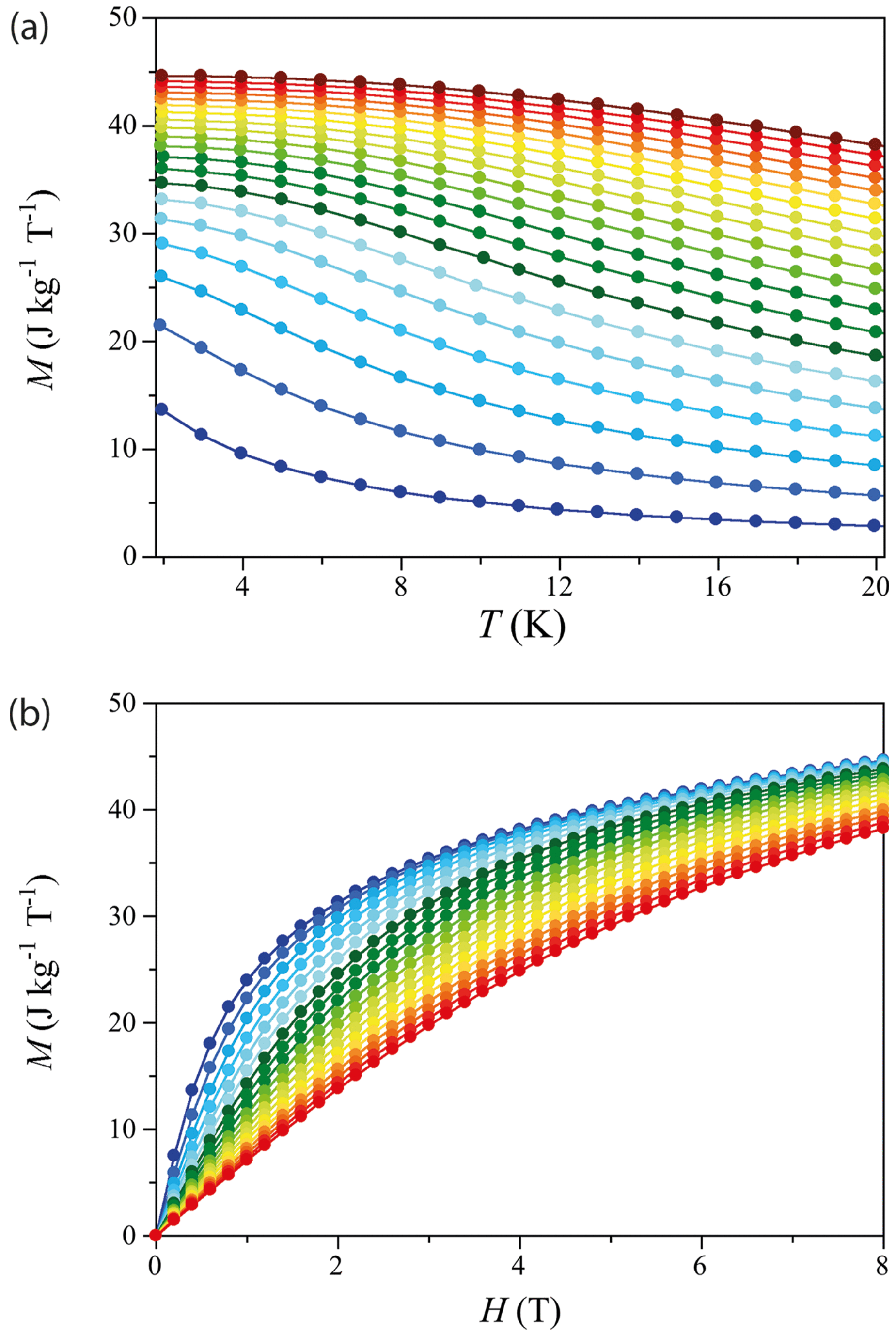

Figure S12. (a) Temperature dependence of M for **1** in the field range 0–8 T (from dark blue to dark red in steps of 0.4 T). (b) Field dependence of M for **1** in the temperature range 2–20 K (from dark blue to red in steps of 1 K). The solid lines are only eye-guides.

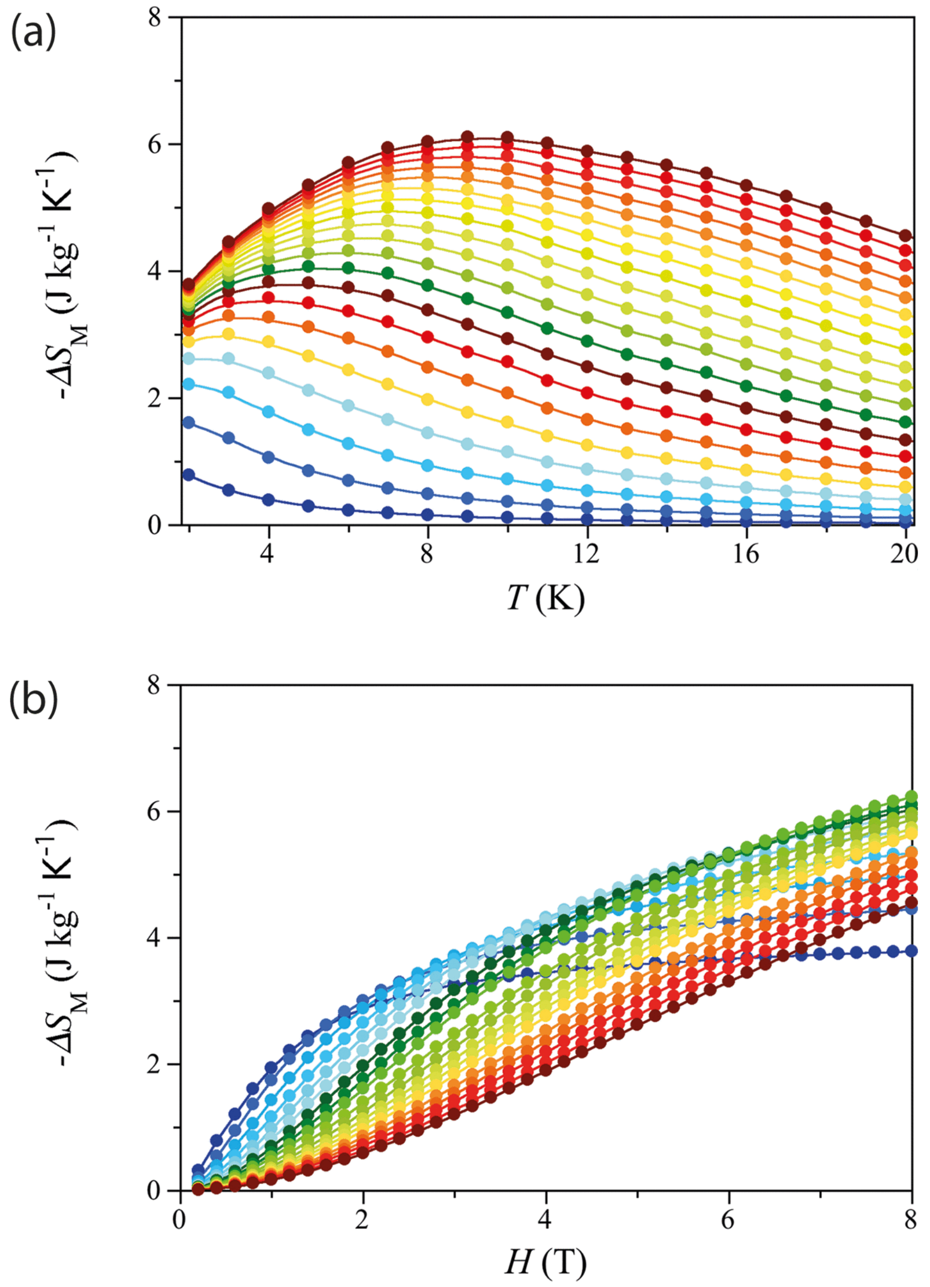

Figure S13. (a) Temperature dependence of $-\Delta S_M$ for **1** in the field range 0–8 T (from dark blue to dark red in steps of 0.4 T). (b) Field dependence of $-\Delta S_M$ in the temperature range 2–20 K (from dark blue to dark red in steps of 1 K). The solid lines are only eye-guides.
